# Real-World Evidence of Improved Glycemic Control in People with Diabetes Using a Bluetooth-Connected Blood Glucose Meter with a Mobile Diabetes Management App

**DOI:** 10.1089/dia.2022.0134

**Published:** 2022-09-29

**Authors:** Mike Grady, Hilary Cameron, Amey Bhatiker, Elizabeth Holt, Oliver Schnell

**Affiliations:** ^1^ LifeScan Scotland Ltd., Beechwood Park North, Inverness, United Kingdom.; ^2^ LifeScan, Malvern, Pennsylvania, USA.; ^3^ Forschergruppe Diabetes e.V., Helmholz Center Munich, Neuherberg (Munich), Germany.

**Keywords:** Real world evidence, Digital ecosystem, Diabetes application, Blood glucose monitor, Readings in-range

## Abstract

**Background:**

The OneTouch Verio Reflect (OTVR) meter provides ColorSure Dynamic Range Indicator (DCRI) and Blood Sugar Mentor (BSM) features that are complemented by the OneTouch Reveal (OTR) mobile app. We sought to provide real-world evidence that these products support improved glycemic control.

**Methods:**

Anonymised glucose and app analytics were extracted from the LifeScan server for 4154 people with type 1 diabetes (PwT1D) and 13,623 people with type 2 diabetes (PwT2D). Data from their first 14 days were compared with the 14 days before the 90-day time point using paired within-subject differences.

**Results:**

Percentage glucose readings in range (RIR) 70–180 mg/dL improved by +8.1% (from 58% to 66.1%) in PwT1D and by +11.2% (from 72.4% to 83.6%) in PwT2D. Hyperglycemic readings (>180 mg/dL) reduced by −8.5% (from 37.1% to 28.6%) in PwT1D and by −11.3% (from 26.4% to 15.1%) in PwT2D. Mean glucose reduced on average by −14.5 mg/dL (from 174.8 to 160.2 mg/dL) in PwT1D and −18.2 mg/dL (from 157.8 to 139.6 mg/dL) in PwT2D. Glycemic improvement was strongly associated with OTR app engagement. Two to three sessions or 11 to 20 min/week in the app improved readings in range in PwT1D by +7.0% or +8.4%, respectively. Similar engagement trends for glycemic improvement were observed in PwT2D. Proportions of subjects achieving a 5% or 10% improvement in RIR were 46.9%/36.6% for PwT1D and 48.7%/37.7% for PwT2D.

**Conclusions:**

Real-world data from over 17,000 people with diabetes (PWDs) demonstrated significantly improved readings in range and reduced the burden of hyperglycemia in PWDs using the OTVR meter and OTR app.

## Introduction

Traditionally, the clinical community has relied on evidence from randomized controlled trials (RCTs) as the gold standard approach to determine the value of new technologies. However, RCTs tend to provide data from a selected population and do not necessarily reflect a new technology's performance in the broader population.^1^

Real-world evidence (RWE) has been described as evidence regarding the usage, benefits, or risks of a product, derived from analysis of real-world data, and is a powerful way to appraise performance of new technologies in more diverse patient populations.^2^ For example, RWE in people with diabetes (PWDs) found that patient engagement with a blood glucose monitoring (BGM) device, a diabetes application, and a coaching program was associated with improved glycemic control, especially among active users of the program.^3^

RWE from over 2000 highly engaged users (logging data ≥5 days/week) of a mobile health app demonstrated improved average blood glucose (BG) levels over a 6-month period.^4^ Furthermore, data from 4555 members of an employer program using a connected BGM device and real-time coaching showed fewer abnormal glucose excursions over 1 year.^5^ The value of retrospective analysis of data has also been demonstrated for continuous glucose monitoring (CGM) systems.

Retrospective analysis of server data from 35,993 patients using a CGM app demonstrated that highly engaged patients had significantly higher time in range (TIR) than less engaged patients.^6^ Such observations are complemented by data from 22,949 Spanish users of flash glucose monitoring, where more frequent engagement (scanning frequency) translated to reduced time in hyperglycemic ranges and more TIR.^7^

Studies have also compared BGM with CGM in terms of outcomes. The Dexcom MOBILE study in people with type 2 diabetes (PwT2D) found that usage of the LifeScan Verio Flex BGM device in combination with the OneTouch Reveal (OTR) mobile app resulted in lowered hemoglobin A1c (A1c) by −0.6%, with the G6 CGM system lowering A1c by an additional −0.4%.^8^

Furthermore, PwT2D initiating structured testing with a variety of BGM devices had similar A1c and TIR improvements among subjects who started using CGM.^9^ In people with type 1 diabetes (PwT1D), improved A1c levels have also been observed when subjects used a Bluetooth-connected BGM device with a diabetes app compared with subjects remaining on their current BGM devices who also did not receive the diabetes app.^10^

Real-world survey data can also provide insights into patient management strategies. A survey of 355 health care professionals (HCPs) found that over 95% agreed that the OneTouch Verio Reflect (OTVR) glucose meter helped their patients understand when results were low, in range, or high, and over 90% agreed that the way the meter displayed information would make patients more inclined to act on results.^11^

A follow-up survey of 353 different HCPs found that endocrinologists, primary care physicians, and diabetes nurses gave significantly higher rankings for the OTVR meter respecting 13 diabetes clinical guidelines compared with three other BGM devices.^12^ This analysis uses RWE from app uploads to confirm the positive survey data from HCPs to demonstrate the impact of the OTVR meter in combination with the OTR mobile app on glycemic control in PWDs.

## Methods

**FIG. 1.**
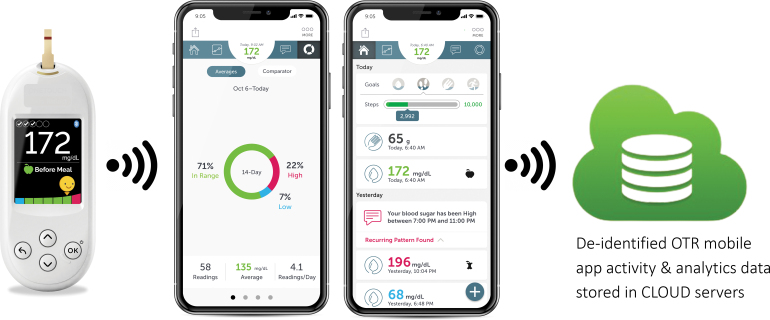
OTVR meter, OTR app, and CLOUD data collection. CLOUD; OTR, OneTouch Reveal; OTVR, OneTouch Verio Reflect.

The Bluetooth^©^ technology in OTVR meters allows PWDs to sync their meters to the OTR mobile app (Fig. 1). Data automatically uploaded from the OTR app are stored within a live Oracle Database hosted on Amazon RDS, encompassing OTR app data from 22 countries. Before the data from Amazon RDS for Oracle are copied to a storage service called Amazon S3, personally identifiable information is removed.

Data from Amazon S3 are queried using the Amazon analytics service, AWS Athena. Data from all countries are fetched by querying AWS Athena and loaded to an AWS Redshift cluster in the United States. PostgreSQL identified subjects who used both OTVR and OTR and who had performed at least 180 readings over the first 180 days of using OTR.

This analysis request automatically fetched meter BG readings for users who registered between June 18, 2018, and May 23, 2021. Subject data were assigned a unique alphanumeric identification (ID), and this was the only identifier present in AWS Redshift and Athena. The usage of an ID is important in terms of user privacy and data protection. Athena also provided diabetes type and an association with the glucose data per subject ID.

OTR app analytics (e.g., time spent, number of sessions, and screens viewed in the OTR app) were also available in the servers and could be correlated with the BG data from individual subject IDs. The current analysis dataset is specific to new users of the OTVR meter and OTR app and focused on the first 90 days after PWDs registered their meter and app.

### Statistical analyses

All analyses were performed separately for each diabetes type. The following glycemic indicators were used for grouping all BG readings: low (<70 mg/dL), in range (70–180 mg/dL), and high (>180 mg/dL). The number of days from when a subject first started using the OTVR meter with the OTR app was determined and time windows were created for baseline (first 14 days) and 90 days (last 14 days before the 90-day time point).

Only subjects with data available within both time windows were retained for analysis to enable pairwise comparisons between their starting and ending values. For each subject, the mean BG and percentage of readings within each of the glycemic indicator categories were calculated for baseline and 90-day time windows and the within-subject changes from baseline were determined.

OTR app analytics data were used to further investigate the changes from baseline to 90 days. For each subject, the number of sessions and time spent on the OTR app per week were categorized as follows: <1 session per week, 1, 2–3, 4–10, and >10 sessions per week and ≤2 min/week, 3–5, 6–10, 11–20, 21–60, and >60 min/week.

All statistical comparisons, between baseline and 90 days, were performed by paired-sample *t*-tests using IBM SPSS Statistics 21 and Minitab 20.

## Results

This analysis includes data from 4154 PwT1D and 13,623 PwT2D who had BG data available in the server for both the first 14 days using the OTVR meter with the OTR app (baseline) and the 14 days before the 90-day usage (90 days). A total of 2,069,390 and 4,550,601 BG readings were available for the 4154 PwT1D and 13,623 with PwT2D, respectively, over the entire 90 days.

### Overall changes in glycemic control

In PwT1D and PwT2D, average BG reduced by −14.5 and −18.2 mg/dL, respectively, comparing baseline with 90 days. Readings in range (RIR) improved significantly by +8.1% (58.0% to 66.1%) and +11.2% (72.4% to 83.6%) in PwT1D and PwT2D, respectively. Hyperglycemic readings were reduced by −8.5% (37.1% to 28.6%) and −11.3% (26.4% to 15.1%) in PwT1D and PwT2D, respectively, mirroring the positive changes in readings in range. Hypoglycemic readings increased marginally, but not clinically significantly, by +0.4% (4.8% to 5.3%) in PwT1D.

The proportion of hypoglycemic readings in PwT2D was lower than in PwT1D and remained essentially unchanged in PwT2D comparing baseline with 90 days, increasing by only +0.1% (from 1.2% to 1.3%). Baseline BG test frequency was higher for PwT1D than PwT2D (3.9 vs. 2.7 tests per day). Both groups significantly reduced their frequency of BG testing by an average of 0.7 tests per day comparing baseline with 90-day use of the meter and app (Table 1).

**Table 1.** Summary of Aggregated Glycemic Data Over 90 Days

People with T1D (*N* = 4154)	Baseline (first 14 days)	90 Days (last 14 days)	Change (baseline to 90 days*^a^*)	95% CI for change (baseline to 90 days*^a^*)
Mean glucose, mg/dL	174.8	160.2	−14.5 mg/dL	−16.2 to −12.9 mg/dL
% <70 mg/dL	4.8	5.3	+0.4%	+0.2% to +0.7%
% Readings in range (70–180 mg/dL)	58.0	66.1	+8.1%	+7.3% to +8.8%
% >180 mg/dL	37.1	28.6	−8.5%	−9.3% to −7.7%
Average test frequency (per day)	3.9	3.1	−0.71	−0.77 to −0.65

People with T2D (*N* = 13,623)	Baseline (first 14 days)	90 Days (last 14 days)	Change (baseline to 90 days*^a^*)	95% CI for change (baseline to 90 days*^a^*)
Mean glucose, mg/dL	157.8	139.6	−18.2 mg/dL	−18.9 to −17.5 mg/dL
% <70 mg/dL	1.2	1.3	+0.1%	+0.06% to +0.21%
% Readings in range (70–180 mg/dL)	72.4	83.6	+11.2%	+10.8% to +11.6%
% >180 mg/dL	26.4	15.1	−11.3%	−11.8% to −10.9%
Average test frequency (per day)	2.7	2.0	−0.67	−0.69 to −0.64

^a^ Average of within-subject paired differences. All changes were statistically significant at *P* < 0.0005.

CI, confidence interval; T1D, type 1 diabetes; T2D, type 2 diabetes.

All glycemic changes, including changes in testing frequency, were statistically significant at the *P* < 0.0005 level. Further analysis of the entire dataset found that 46.9% (1948 of 4154) of PwT1D improved RIR by >5% and 36.6% (1519 of 4154) improved RIR by >10%. Similarly, 48.7% (6638 of 13,623) of PwT2D improved RIR by >5% and 37.7% (5133 of 13,623) improved RIR by >10%.

### Effect of the number of OTR app sessions on glycemic control

Significant reductions in mean glucose were observed in PwT1D engaging in at least one app session per week (−5.8 mg/dL) over the 90 days, with the highest reduction in mean BG seen in those subjects engaging in 4 to 10 sessions (−21.1 mg/dL) and >10 sessions per week (−25.5 mg/dL). Clinically meaningful improvements in readings in range were seen in PwT1D conducting at least 2 to 3 app sessions per week (+7.0%), and this increased to +13.2% in those conducting >10 sessions per week.

**FIG. 2.**
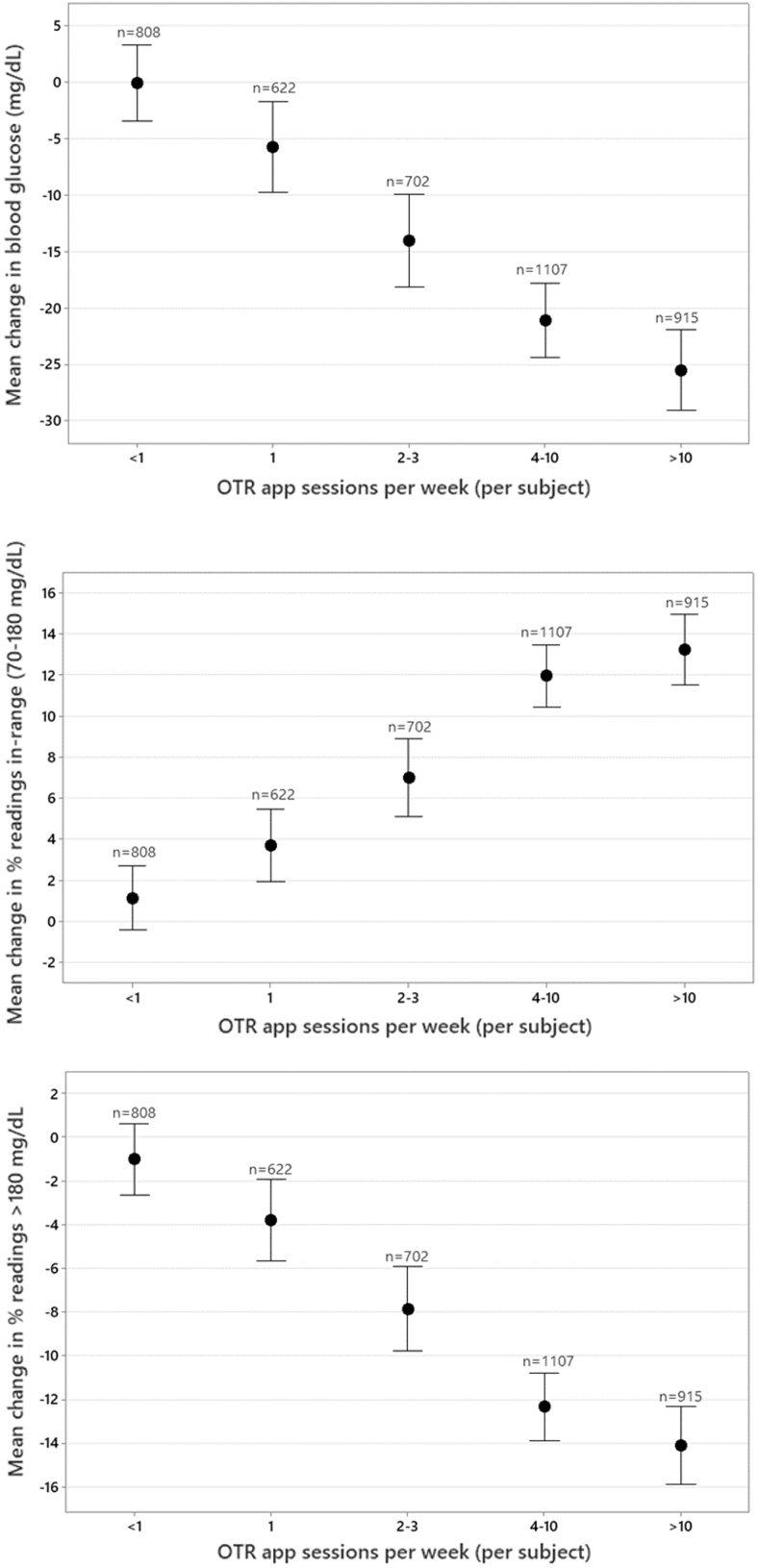
Effect of the number of app sessions on glycemic changes in people with type 1 diabetes.

Improvements in readings in range were mirrored by concomitant reductions in hyperglycemia in PwT1D, with clinically significant reductions in hyperglycemia observed in PwT1D engaging in at least 2 to 3 app sessions per week (-7.9%), increasing to −14.1% in those engaging in >10 sessions per week. Significant improvements in mean glucose, readings in range, and hyperglycemia were not observed in PwT1D engaging in less than one app session per week (Table 2 and Fig. 2).

**Table 2.** Effect of the Number of App Sessions on Glycemic Changes in People with Diabetes

OTR app sessions (per week)	<1 Session		1 Session		2 to 3 Sessions		4 to 10 Sessions		>10 Sessions	
Diabetes type	T1D (*n* = 808)	T2D (*n* = 1673)	T1D (*n* = 622)	T2D (*n* = 1490)	T1D (*n* = 702)	T2D (*n* = 2090)	T1D (*n* = 1107)	T2D (*n* = 4901)	T1D (*n* = 915)	T2D (*n* = 3469)
Mean glucose (mg/dL)	−0.11	−8.69	−5.76	−17.25	−14.07	−17.69	−21.11	−19.31	−25.55	−21.88
% Readings in range (70–180 mg/dL)	+1.15	+5.12	+3.71	+10.37	+7.01	+11.42	+11.95	+12.31	+13.23	+12.81
% Hyperglycemic readings (>180 mg/dL)	−1.03	−5.39	−3.81	−10.49	−7.87	−11.46	−12.34	−12.42	−14.11	−13.00
Average test frequency	3.15	2.12	3.24	2.07	3.20	2.03	2.96	1.91	3.87	2.59

OTR, OneTouch Reveal.

In our larger dataset of 13,623 PwT2D, we observed very similar levels of improvement in mean glucose and readings in range and reductions in hyperglycemia as those recorded in PwT1D. However, in contrast to PwT1D, PwT2D who engaged in less than one app session per week did significantly improve mean glucose (−8.7 mg/dL) and readings in range (+5.1%) and had meaningful reductions in hyperglycemia of −5.4% (Table 2 and Fig. 4).

It is notable that BG testing frequency was significantly higher in both PwT1D and PwT2D who engaged in the highest number of app sessions per week compared with those subjects engaging in the fewest app sessions per week, but this was consistent across the full 90 days.

In fact, the testing frequency declined in all groups across the number of app sessions per week and across time spent on the app, so while the more engaged subjects did tend to test more frequently than the least engaged subjects, there was not an increase in testing frequency over the 90 days to explain the improvement in measures of glycemia in any of the groups.

### Effect of time spent on the OTR app on glycemic control

Significant reductions in mean glucose (−5.5 mg/dL) were observed in PwT1D who spent at least 6 to 10 min/week reviewing and/or adding data to the app, with the highest reductions in mean glucose recorded in those spending 21 to 60 min (−19.2 mg/dL) and >60 min/week (−29.4 mg/dL) in the diabetes app. Clinically meaningful improvements in readings in range were seen in PwT1D who spent 11 to 20 min/week (+8.4%) in the app, increasing to +14.1% in those spending >60 min/week.

**FIG. 3.**
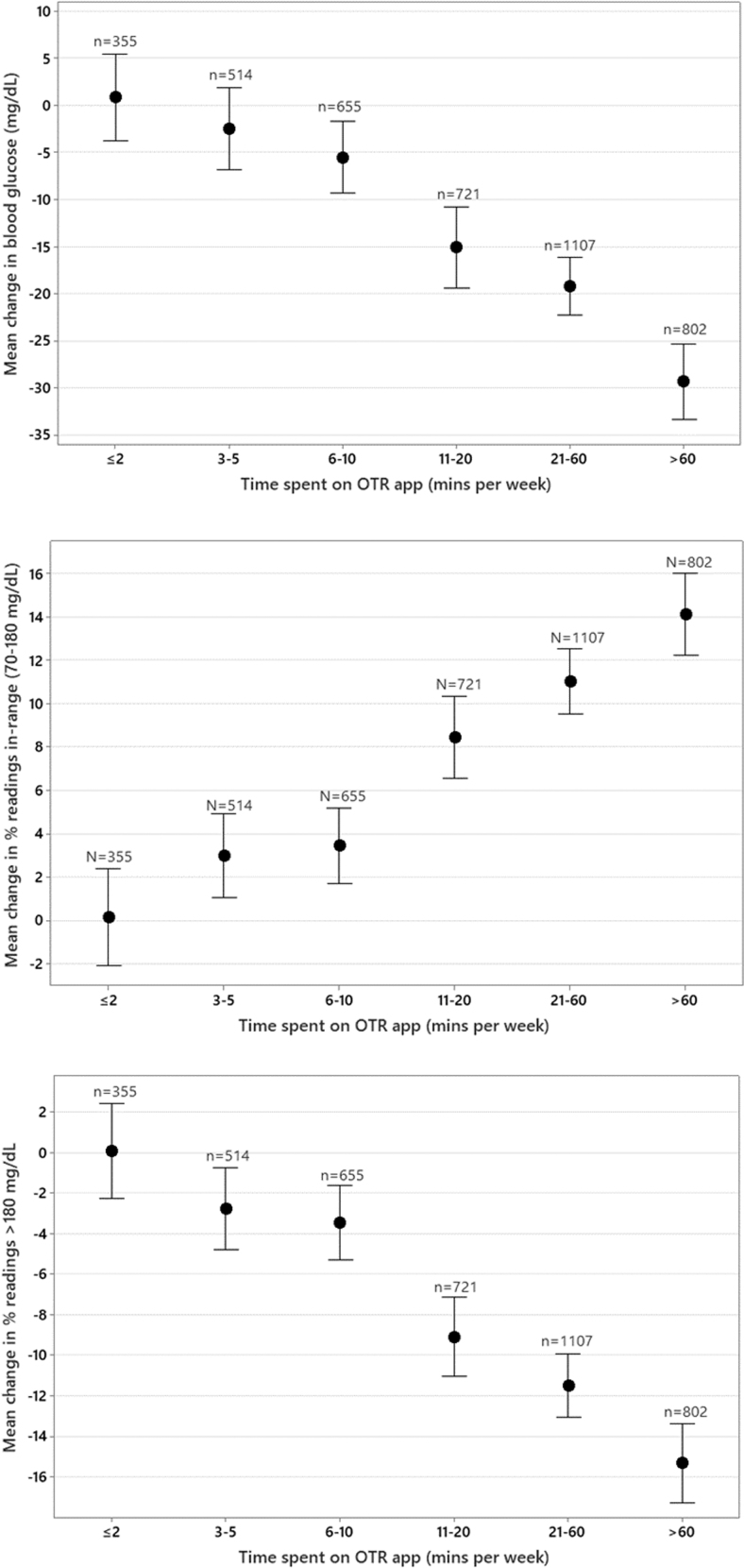
Effect of time spent in app sessions on glycemic changes in people with type 1 diabetes.

Improvements in readings in range were largely explained and mirrored by reductions in hyperglycemia in these PwT1D. Significant reductions in hyperglycemia were observed in PwT1D spending 11 to 20 min/week in the app (−9.1%), increasing to −15.3% in those spending >60 min/week in the app. Clinically significant improvements in mean glucose, readings in range, or hyperglycemia were not observed in PwT1D spending <11 min/week in the app. (Table 3 and Fig. 3).

**FIG. 4.**
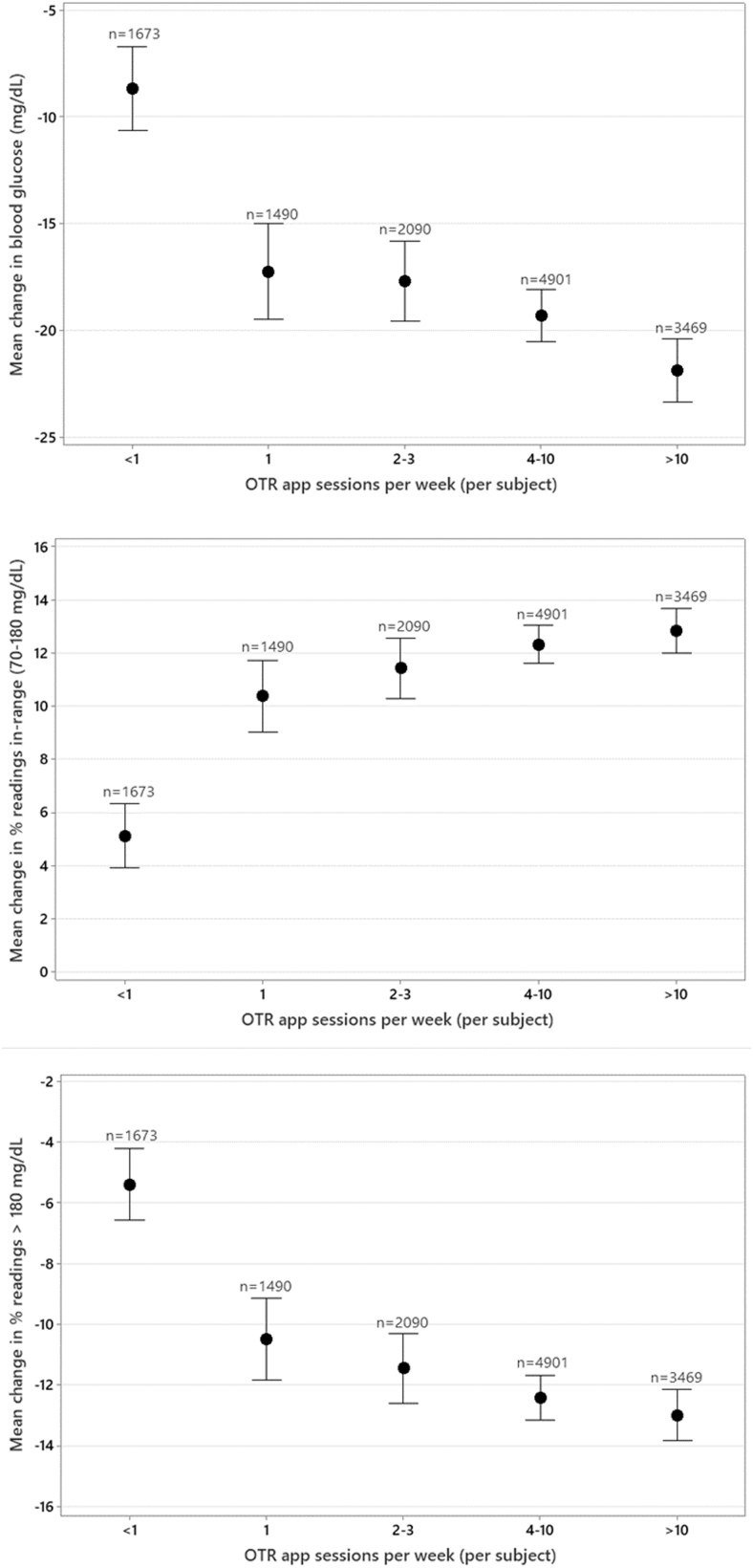
Effect of the number of app sessions on glycemic changes in people with type 2 diabetes.

**Table 3.** Effect of Time Spent on the App on Glycemic Changes in People with Diabetes

Time on app (per week)	≤2 min		3 to 5 min		6 to 10 min		11 to 20 min		21 to 60 min		>60 min	
Diabetes type	T1D (*n* = 355)	T2D (*n* = 849)	T1D (*n* = 514)	T2D (*n* = 1265)	T1D (*n* = 655)	T2D (*n* = 1728)	T1D (*n* = 721)	T2D (*n* = 2439)	T1D (*n* = 1107)	T2D (*n* = 4701)	T1D (*n* = 802)	T2D (*n* = 2641)
Mean glucose (mg/dL)	+0.84	−5.05	−2.48	−14.20	−5.53	−17.79	−15.11	−17.81	−19.20	−19.60	−29.36	−22.40
% Readings in range (70–180 mg/dL)	+0.16	+3.00	+2.98	+8.13	+3.45	+10.54	+8.45	+11.87	+11.03	+12.18	+14.13	+13.40
% Hyperglycemic readings (>180 mg/dL)	+0.05	−3.12	−2.78	−8.43	−3.48	−10.77	−9.12	−11.76	−11.51	−12.35	−15.33	−13.57
Average test frequency	3.02	2.15	3.15	1.99	3.19	2.02	3.05	1.97	3.04	2.04	4.08	2.66

**FIG. 5.**
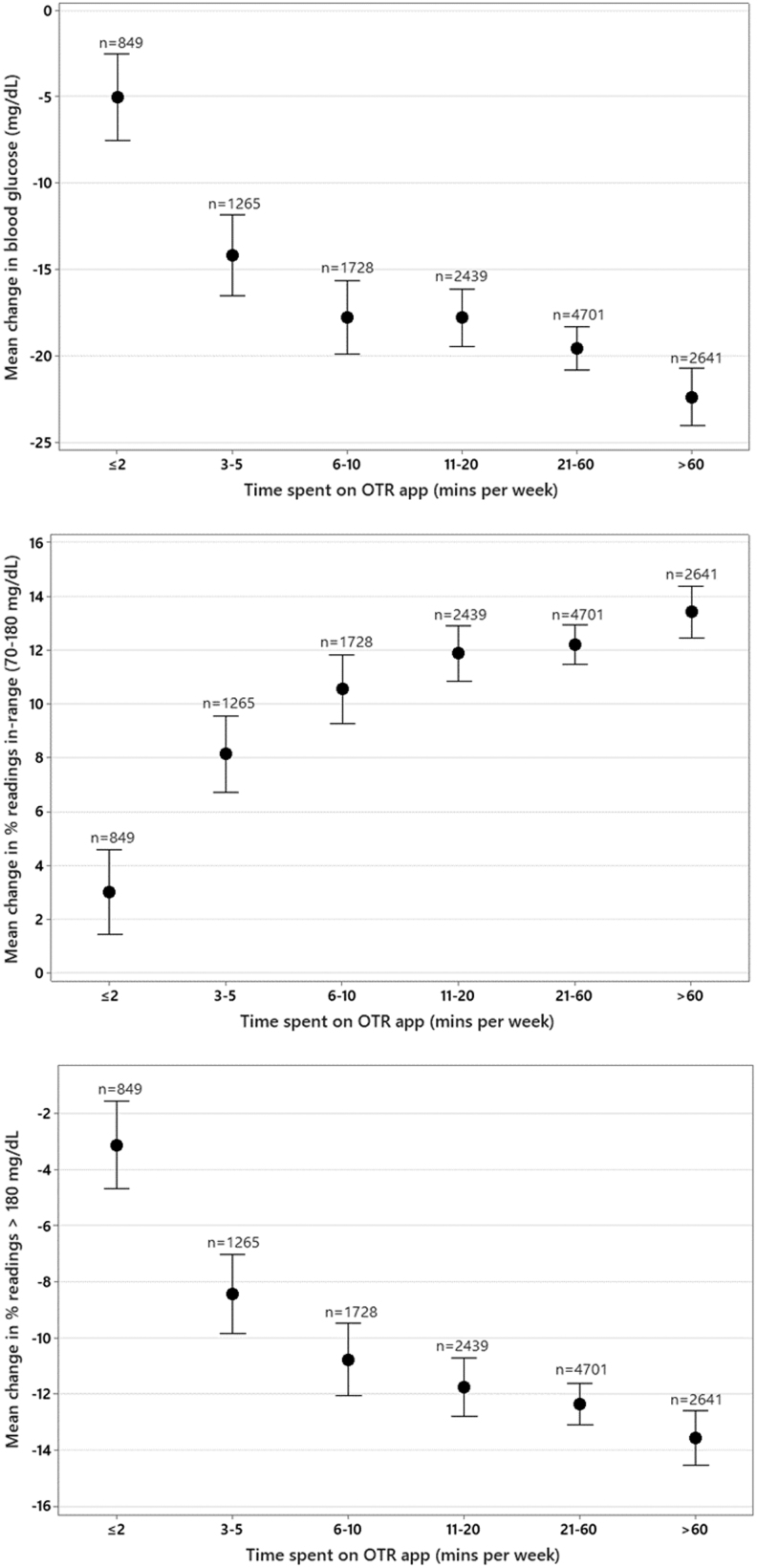
Effect of time spent in app sessions on glycemic changes in people with type 2 diabetes.

In our dataset of PwT2D who spent at least 11 to over 60 min/week in the app, we observed very similar levels of improvement in mean glucose, readings in range, and hyperglycemia as those found in PwT1D. However, in contrast to PwT1D, PwT2D who spent as little as 3 to 5 min/week in the app did achieve significant improvements in mean glucose (−14.2 mg/dL) and readings in range (+8.1%) and significant reductions in hyperglycemia of −8.4% (Table 3 and Fig. 5).

The level of app engagement per week by PwT2D was a surprise finding. In keeping with data on the impact of the number of app sessions on testing frequency, a similar picture emerged between BG test frequency and time spent on the app, with significantly higher BG testing observed in subjects spending the most time in the app compared with those spending the least time on the app.

## Discussion

This real-world data analysis shows evidence for improved glycemic control in PwT1D or PwT2D who started using a specific Bluetooth-connected blood glucose monitoring (BGM) device with the OTR diabetes management app. We described specific improvements in glycemic control as a change in readings in range (RIR) rather than the now familiar term “time in range” (TIR), which has gained widespread recognition in the arena of CGM using devices that legitimately give a report on TIRs given that they measure interstitial glucose every 1 to 5 min.

In contrast, our analysis is based on episodic, capillary BG testing with a BGM device, so we chose the term “readings in range” (RIR) to denote BGM readings from 70 to 180 mg/dL, which are the commonly accepted criteria for in range data.^13^ Keynote articles based on CGM data highlight that a predicted (estimated) A1c level based on TIR or on time spent >180 mg/dL has essentially the same degree of precision and reliability as an estimated A1c level based on mean glucose.

These CGM articles also demonstrated that even a small percentage change in TIR can be clinically meaningful, with a 5% increase in TIR or a 5% decrease in time >180 mg/dL representing an extra 1.2 h in range each day. Furthermore, a 5% change in either metric would equate to a clinically meaningful improvement in A1c of ≥0.4%.^14,15^

Given that we do not have the same quantity of readings per patient for BGM users compared with CGM users, we determined it was more appropriate to explore trends in glycemic control, and there would be more weight to our findings, by utilizing a far larger dataset of PWDs using BGM than is common in most RCTs with CGM. For context, the clinical findings in the two landmark DIAMOND studies (using Dexcom CGM) were based on 105 PwT1D and 79 PwT2D, respectively.^16,17^ Clearly, irrespective of scale, RCTs are the gold standard with prespecified endpoints powered to demonstrate significance.

To bolster our collective BGM and OTR app findings and glycemic trends, we analyzed real-world data from 4145 PwT1D and 13,623 PwT2D and found that readings in range improved on average by 8% and 11%, respectively, over 90 days. This clinically significant improvement in RIR, which is above the 5% threshold for a clinically meaningful change, was not associated with an increase in BGM test frequency.

In fact, test frequency was significantly higher in the first 14 days that PWDs experienced their new meter and app compared with the last 14 days before day 90, suggesting that there is an initial discovery period for PWDs after exposure to the new features and insights from the OTVR BGM and OTR app.

Our summary glucose data observed a small but statistically detectable increase in the proportion of hypoglycemic readings for PwT1D (but not PwT2D), which could be explained by tighter glycemic control, although our improved readings in range seem fully accounted for by reductions in hyperglycemia.

The OTVR meter has a ColorSure Dynamic Range Indicator (DCRI) (Fig. 1) and uses a variety of automated algorithms to display on-screen insights and trends in an engaging way. Collectively, this guidance, insight and encouragement are described as the Blood Sugar Mentor (BSM) features.

It is plausible that the improved RIR data we observed resulted from new behaviors, decisions, and/or actions based on the DCRI and BSM, supported by information shown in the app, such as the personal journey shown on the app home page (Fig. 1). The home pages also show recent readings, identify glycemic patterns, and enable PWDs to tag readings to give context to daily events, such as food intake or taking medications.

Evidence for the ability of a similar color range indicator to improve glycemic control was previously shown in an RCT comparing PWDs using a meter with a color range indicator with PWDs who remained on BGM devices without color range indicators.^18^ The more advanced OTVR meter in our RWE analysis combines color range indication with BSM features, so we surmise that a synergistic benefit in terms of improving glycemic control explains the observed trends.

We also know that HCPs in five countries ranked the OTVR meter as the best meter to enable them to deliver against clinical guidance goals in comparison with other leading BGM devices,^12^ with 80% of HCPs agreeing that OTVR was the best meter to help patients understand their numbers to help them stay in range. A factor in the success of any technology is the level of patient engagement, and this association is equally true for BGM, CGM, or diabetes apps.

An RWE analysis of CGM observed that PWDs who seldom interacted with their device (lower scanning frequency) had poorer estimated A1c outcomes,^19^ and a recent study in 461 PWDs using the Contour Diabetes app reported that 40% stopped using the app before 16 weeks.^20^ We have internal data from our data lake suggesting that about 10% of PWDs stop using the Reveal app based on an evaluation of app uploads over a 1-year time frame (data on file).

Therefore, we anticipated different levels of engagement in our large dataset and sought to describe how engagement impacted outcomes. We found a clear association between more app sessions or more time spent on the OTR app and higher levels of RIR and fewer readings >180 mg/dL (hyperglycemia). The improvement in percentage of RIR was 0% and 3% for PwT1D and PwT2D, respectively, who spent ≤2 min/week in the OTR app compared with improvements of 14% and 13% for PwT1D and PwT2D, respectively, who spent >60 min/week in the OTR app.

The engagement level, as a function of sessions completed in the app, found a similar association, with RIR improving by 1% to 13% and 5% to 13% in PwT1D and PwT2D, respectively, who did <1 session to >10 app sessions per week. It was noteworthy that PwT2D who took the time to do even one session (or 3–5 min/week) in the app made significant improvements, whereas for PwT1D, there was a more gradual stepwise improvement in RIR, implying that more time and effort were required to achieve clinical improvements.

It may be that prior diabetes knowledge or engagement in our subjects with T2D, who may have previously been using systems without guidance features, was low enough that even a small incremental review and reflection on their glucose data were sufficient to make a clinical impact on their diabetes management. Arguably, PwT1D may have a higher baseline comprehension of how daily factors and decisions affect their glucose profiles such that significantly more time and effort exploring the guidance and insights offered by the meter and app were required to elicit glycemic improvements.

Although the BG test frequency reduced over time in our subjects, we did identify that PwT1D and PwT2D with the highest level of app engagement tested more often than PWDs in the least engaged app category. This higher frequency of testing may partially explain the improved glycemic control in the most engaged groups of patients, but because these groups decreased testing frequency over the 90 days, the testing frequency does not fully explain the glycemic improvements.

### Study limitations

With RWE analyses, there are often limitations in terms of our knowledge of the subjects' medical history, adherence to and/or changes in medications during the study period, or the clinical goals set by their HCPs. Furthermore, we cannot verify the types of HCPs, how they used the meter or app data (or A1c) to adjust therapy, or how often therapy changes were made, and it is unclear if specific subjects were offered CGM during this time frame while continuing to use the new BGM device.

Given that the time frame was 90 days, it is plausible (although unlikely) that many subjects changed their HCP, which could also influence clinical outcomes. Our dataset may also contain a proportion of PWDs new to self-monitoring and these subjects could be expected to improve glycemic control, irrespective of the BGM system they received. With respect to this BGM dataset and our description of RIR, we are cognizant that BGM data are episodic, rarely collected during the night, and for the most part (and especially for PwT2D), predominantly premeal BG testing data.

The lack of data on test regimens (such as whether PWDs changed pre- or postmeal glucose test habits or tested in response to hypoglycemia) may also influence RIR. Furthermore, we did not have access to the baseline A1c levels of study subjects, which could influence the extent of any glycemic improvement, particularly for subjects already with good glycemic control.

## Conclusions

Real-world data from over 17,000 PWDs demonstrated a clinically significant improvement in BG readings in range, with a proportionate and clinically significant reduction in hyperglycemic readings in PwT1D and PwT2D using a Bluetooth-connected BG meter with a mobile diabetes management app.

The improvements in glycemic control were enhanced by, and strongly correlated with, increasing engagement in terms of time spent or number of sessions interacting with the mobile diabetes management app.
